# Building Sustainable Supply Chains for Organizations Based on QFD: A Case Study

**DOI:** 10.3390/ijerph15122834

**Published:** 2018-12-12

**Authors:** Zhifeng Wu, Senjing Zhai, Jiangtao Hong, Yibin Zhang, Keren Shi

**Affiliations:** 1 Institute of Logistics Science and Engineering, Shanghai Maritime University, Shanghai 201306, China; wu_zhifeng@suibe.edu.cn; 2 International Business School, Shanghai University of International Business and Economics, Shanghai 201620, China; hongjiangtao@suibe.edu.cn (J.H.); qiuqiu@suibe.edu.cn (K.S.); 3 School of Business Administration, Shanghai Lixin University of Accounting and Finance, Shanghai 201620, China; zhangyb@lixin.edu.cn

**Keywords:** sustainable supply chain, quality function deployment, house of quality

## Abstract

Environmental protection has been increasingly emphasized by stakeholders, including social organizations, the government, and the public. As a result, building a sustainable supply chain has now become a part of social corporate responsibility as well as a challenge for firms, including small and medium-sized enterprises (SMEs). Taking a chemical enterprise (called enterprise C, in this paper) as an example, this paper uses quality function deployment (QFD) techniques and sets up a house of quality (HOQ) to investigate how a SME can achieve sustainable supply chain management. Specifically, in this paper we build a performance measurement system focusing on economic performance, environmental performance, and social performance. These three types of performance measure, in turn, include fifty-nine secondary indicators. Furthermore, an analytic hierarchy process (AHP) has been adopted to calculate the weight of each indicator. Benchmarking has also been used, to determine how much the enterprise should improve on each indicator. Based on the HOQ model, we conclude that avoiding waste, recycling resources, and sustainable exploitation are most important internal abilities of enterprise C.

## 1. Introduction

Small and medium-sized enterprises (SMEs) contribute greatly to the economy of many countries. However, SMEs are responsible for approximately 70% of global industrial pollution [1]. A limited number of SMEs have sustainability practices and goals [2]. This might be due to the fact that SMEs, with their relatively limited resources (compared to large firms), face more challenges in terms of sustainability issues.

Many scholars have examined the sustainable development issues of SMEs from the perspective of strategy and operations management. Pedersen and Loucks et al. pointed out that sustainable development strategies and approaches need to be innovated to consider the various characteristics of SMEs, as replicating exactly what large firms are doing in sustainability have been found inappropriate [3,4]. Thanki argued that SMEs should not focus just on operational performance, but also on the integration of sustainability practices in their supply chains [5]. Burke and Gaughran suggested that SMEs need to develop practical approaches or tools, required to implement and manage sustainability aspects in their operations [6]. Therefore, how to identify and establish an appropriate management framework to their sustainable development is a vital issue for SMEs.

With the increasing prominence of sustainable development in developing countries, a matter of concern is the trend of polluting industries moving from developed to developing countries. Being the biggest developing country in the world, China has suffered from serious industrial pollution, one of the main sources of which is from SMEs. The pollution load (i.e., the quantity of pollutants present in an environment) accounts for about 50% of industrial pollution, and has an increasing trend. Although laws and regulations have been promulgated by Chinese government to control the emission of industrial waste and poisonous gases, these alone have not been sufficient to solve the problem fundamentally. The aim of realizing more environmentally friendly development requires enterprises, especially SMEs, to reform their business philosophy and increase investment in sustainable development, in the long run.

In the case of the domestic market in China gradually becoming mature, the competition between enterprises has changed from firm-wide to supply chain-wide. Therefore, how to build a sustainable supply chain and integrate a new management method into daily operations have become the main points for enterprises. However, most Chinese SMEs have limited knowledge of sustainable supply chains. They have gotten used to always putting economic performance at the core attention of their business. Currently, it’s of great importance for these SMEs to balance the input of establishing the sustainable supply chain and the economic returns. As a result, the solution should achieve the coordination of economic performance, social performance, and environmental performance at the same time.

The above-mentioned discussion identifies that there exists a gap in the literature to show how SMEs will build their sustainable supply chains. In addition, there exists another research gap in how to guide sustainable supply chain management (SSCM) in developing countries, toward its strategic implementation. In order to fill these two research gaps, this study presents a novel integrated model of supply chain management for SMEs, by taking sustainability requirements in the Chinese context. This is achieved by using the quality function deployment (QFD) method, combining an analytic hierarchy process (AHP) and benchmarking for guiding supply chain design in SMEs. An analysis of a cement manufacturing enterprise (called enterprise C, in this paper), which is a representative SME, is conducted to illustrate and validate our approach.

The rest of the paper is organized as follows: Section 2 explains the concept and motivations of SSCM, and the obstacles to its implementation. Section 3 illustrates the research methodology of this paper. Section 4 demonstrates the case study. The last section concludes, discusses the limitations of this paper, and proposes future research directions.

## 2. Literature Review

Over the past two decades, supply chain management (SCM) practices among leading organizations have addressed not only economic concerns, but also environmental and social concerns [7]. Sustainable supply chain management takes into consideration the sustainability of economy, environment, and society at the time of designing and optimizing the supply chain [8,9,10]. As Dubey et al. [10] summed it up, SSCM can be understood as SCM focused on maintaining environmental, economic, and social stability for long-term sustainable growth. Under the notion of SSCM, current research on economic sustainability focuses on a healthy cash flow, good profit margins and a proper return on investment, business performance improvement, and competitive advantage [11,12]. The literature on environmental sustainability pays more attention to energy resource utilization, product recycling, green purchasing, and reduction of emissions, which has created a research stream known as green supply chain management [13,14,15,16,17]. The literature addressing social sustainability investigates various social issues, such as liability to shareholders, employee welfare, prevention of corruption, social image, responsibilities towards the community, and occupational health and safety, which has created a research stream known as socially responsible supply chain management [18,19,20,21].

The implementation of SSCM is not only affected by pressure, but also by impetus. Enterprises engaged in SSCM practices have been under pressure from consumers who are becoming more and more aware of the global sustainability concerns, stakeholders (such as workers, investors, suppliers and unions) [22,23], and legal requirements [24]. Furthermore, some organizations are motivated by the concerns about improving reputation, strategic collaboration, enabling information technologies, logistics optimization, continuous improvement, and acquiring more competitive advantage [23,24].

Although the numerous potential benefits of implementing SSCM have been realized by many enterprises, SSCM practices are still not widely implemented in reality [25,26,27]. There are still huge obstacles faced by enterprises. The regular challenges are: The substantial investments necessary, the lack of green resources, complexity due to the extended scope of SSCM, and mindset and cultural changes, as well as the uncertainties involved [28,29,30]. It requires a lot of effort in management, cooperation, collaboration, controlling, monitoring, and evaluation to implement SSCM practices [31]. Giménez and Lourenço [31] concluded that enterprises need to adopt collaborative practices with their supply chain partners to implement SSCM, which, in turn, highlights the importance of Information System (IS) or Information Technology (IT) to facilitate the required collaboration. Moreover, the increased complexity in measuring outcomes implies a need to have information provision and transparency within and across organizational boundaries, for monitoring and evaluating performance [32].

In addition, supply chains in emerging economies are facing more barriers to sustainability than those operating in developed countries [33,34]. Research focused on emerging countries is still limited, as SSCM practices in these countries are relatively underdeveloped [35,36,37].

In all, it shows that no method in the literature with a clear focus on building sustainable supply chains for SMEs has been articulated yet, in the context of developing countries. This may prevent SMEs in developing countries from applying SSCM to achieve sustainable development. To help solve this problem, this study develops a combined QFD–AHP-benchmarking approach, which can help to achieve sustainable supply chain design and management for SMEs in developing countries.

## 3. Applying QFD to Build a Sustainable Supply Chain Model

This study uses QFD to build a house of quality (HOQ), relates external requirements to internal abilities, and, at last, determines which internal abilities to deploy, with the assistance of AHP and benchmarking.

The analytic hierarchy process (AHP) method is mainly used to calculate the weight of external requirements and internal abilities in the HOQ model. About weight calculation, please see Table 1. For external requirements, economic performance, environmental performance, and social performance are equally weighted. Taking economic performance, for example, reliability, flexibility, responsiveness, and quality use the weights calculated by AHP. In the sub-dimension, each requirement has its own weight in AHP. Therefore, the final weight of each external requirement can be calculated by 1/3 of the economic performance, environmental performance, and social performance, multiplied by the weights of the first dimension and sub-dimension. 

Benchmarking is mainly adopted to conduct the competitive analysis of external requirements and internal abilities in the HOQ model. Through interviews, managers of the enterprise and other experts in the industry score the ability of the enterprise and the benchmark enterprise, according to industry standard. As the result, the improving rate could be obtained as: Improving rate = the score of benchmark enterprise/the score of enterprise

*Step 1. Identify External Requirements*

To build a HOQ, the first (and the most important) step is to identify customer requirements. Through a questionnaire survey of related managers and employees, the customer’s needs and expectations can be identified. Under the evaluation system of a sustainable supply chain, the external requirements are not only economic performance, but also social performance and environmental performance. To evaluate economic performance, this paper employs the four evaluation factors proposed by Vachon & Klassen [38]: Reliability, responsiveness, flexibility, and quality. Five factors, including environmental management, use of resources, pollution, dangerousness, and natural environment, proposed by Murphy [39], are applied to appraise environmental performance. To assess social performance, this paper adopts the factors proposed by Drumwright [40], which are work conditions, human rights, societal commitment, customer issues, and business practices. All the external requirements are shown in Table 2.

*Step 2. Identify Internal Abilities*

To build a sustainable supply chain, an enterprise is required to possess several internal abilities, in order to realize the external requirement [1]. This paper evaluates the internal abilities, according to the process of sourcing, producing, and distribution, so as to make an overall measurement of the enterprise’s daily operations [15]. In the sourcing phase, the cost and quality of the products provided by suppliers play an important role in daily operations. Furthermore, regional and grouped purchasing and central warehouse building will largely increase the sourcing efficiency. Additionally, the sourcing process greatly depends on procurement compliance. In the production phase, sustainable exploitation, avoiding child and forced labor, avoiding waste, and recycling of resources are key internal abilities to realize a sustainable supply chain. In the distribution phase, the ability of computerization helps enterprises improve accuracy and efficiency during distribution, and to achieve overall control of the process. Furthermore, collaborative transportation and adoption of different delivery modes improves the distribution capability. In the actual transport process, reasonable driving time for a driver must be guaranteed, or the risk of fatigue driving may lead to severe consequences.

*Step 3. Relate External Requirements to Internal Abilities*

The third step is to relate external requirements to internal abilities, as follows:

● = 9 highly correlated
▲ = 3 general correlated
○ = 1 poor correlated

*Step 4. Identify the Relationships between Internal Abilities in the Roof of the House*

The relationships between internal abilities are shown on the roof of the houses, which are defined as follows:

● = 9 significant positively correlated
☆ = −9 significant negatively correlated
○ = 3 positively correlated
★ = −3 negatively correlated

*Step 5. Perform a competitive assessment of external requirements*

In this step, it is required to perform a competitive assessment of external requirements. During this step, the difference between enterprises and their competitors in the industry can be clearly quantified, through benchmarking.

*Step 6. Prioritize External Requirements*

This paper adopts AHP to evaluate the importance of each external requirement. Whether the enterprise needs to improve their goods and service, or remain in the current condition, depends on the target score of the benchmarking.

*Step 7. Prioritize Internal Abilities*

Through AHP and benchmarking, the absolute weight and relative weight of the internal abilities are calculated.

*Step 8. Determine Which Internal Abilities to Deploy*

Enterprises should focus more internal resources to, and put more emphasis on, the internal abilities which carry more weight. Through the formal steps in the model, the enterprise determines which internal abilities to deploy, to maximize overall sustainable supply chain performance.

## 4. Case Study

### 4.1. Company Profile

As a cement manufacturer, enterprise C is located in the city of Zhangjiagang, Jiangsu Province, China. The enterprise has three subsidiary factories and its annual output is nine million tons. Although Enterprise C is a SME, the company maintains a good reputation in the industry. To further grow in the next five years, the company determines to increase production and expand market share. However, the company is faced with internal and external pressures. Local government begins to monitor and control pollution intensely. Their partners pay more attention to the company’s public image. Inside the company, personal safety, training mechanisms, and career development are greatly valued by the staff. Therefore, enterprise C determines to build a sustainable supply chain to improve its economic performance, protect the ecosystem, and build a healthy public image. It has been identified that the main problems of building a sustainable supply chain in a cement enterprise are: Sustainable mining and utilization in the upstream, pollution control in the middle stream, and rational planning of distribution and transportation in the downstream.

### 4.2. Application of the Approach

The whole process of applying both QFD and AHP to enterprise C is illustrated in the following steps:

*Step 1. Identify external requirements, internal abilities, and relate external requirements to internal abilities.*

**Figure 1:**
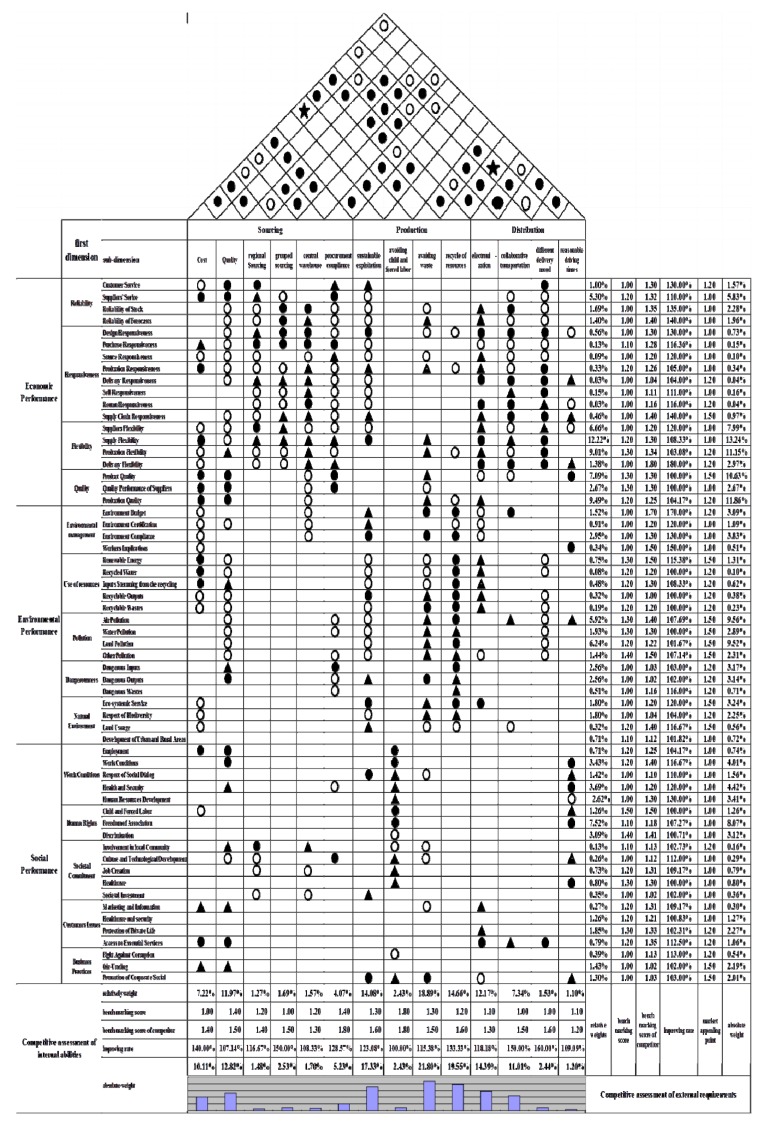
House of quality.

According to existing studies and interviews with managers and experts in the industry, we concluded which external requirements and internal abilities help the company build a sustainable supply chain. Furthermore, their relationships are presented in the HOQ model (shown in Figure 1). Through the relationship matrix, it is clear that among economic performance, environmental performance, and social performance, the sub-dimensions of economic performance have the closest connection with operational competency. In contrast, the sub-dimensions of social performance show a weak relationship with operational competency.

In the relationship matrix of economic performance and internal abilities, the building of a central warehouse, electronic capabilities, and a diverse transportation mode are core capabilities, which can improve quick responsiveness and flexibility while maintaining a high quality of products and service. In this relationship matrix, avoiding child and forced labor, and ensuring reasonable driving time are two internal abilities which have weak connections with economics performance. Additionally, reliable suppliers, quick responsiveness of the supply chain process, flexible procurement, and production could be improved by every process control in the daily operations.

In the relationship matrix of environmental performance and internal abilities, sustainable exploitation, avoiding waste, and recycling of resources could largely satisfy the external requirements of environment management, resource usage, pollution control, and environment protection. In contrast, regional and grouped purchasing, central warehouse building, and avoiding child and forced labor may not have a great contribution in improving social performance. However, the external requirements of renewable energy, recyclable outputs, recyclable wastes, control of dangerous outputs, and ecosystem protection could be satisfied by increase of the input in the production and distribution phases.

In the relationship matrix of social performance and internal abilities, maintaining a high quality, avoiding child and forced labor, and ensuring reasonable driving time would increase the social performance in working condition, human rights, social commitment, customer issues, and business practices. In contrast, regional and grouped purchasing, recycling of resources, and collaborative transportation do not significantly correlate with the social performance. In addition, it is good for the company to value social communication, provide customized service, and improve its sense of social responsibilities.

*Step 2. Identify the relationships between internal abilities of enterprise C.*

According to the distribution of black points, the internal abilities are correlated to each other. For instance, quality of products, avoiding waste, and recycling of resources are positively correlated with cost control. A relatively weak positive-correlated relation can be shown by the distribution of white points. For example, it can be concluded that the improvement of computerization could increase the internal abilities in avoiding waste, ensuring the quality of products in some extent. In general, the cost of products, procurement compliance, recycling of resources, and computerization are the top four internal abilities, which have a strong correlation with other abilities. However, avoiding child and forced labor and ensuring reasonable driving time have a poor connection with other internal abilities.

*Step 3. Perform a competitive assessment of external requirements of enterprise C and prioritize external requirements.*

In this step, all of the external requirements are ranked by absolute weight, which is calculated by relative weight, improving rate, and market appeal point. The relative weights of external requirements are calculated by AHP, which presents the difference of importance between each external requirement, by pairwise comparison. Through benchmarking, the improvement rate of each requirement could be obtained. The market appeal point refers to the key requirement which could greatly increase the customers’ satisfaction. According to the importance of external requirements, it could be divided into:

- Super market appeal point, which the enterprise wishes to satisfy: Weights 1.5.
- Interested market appeal point, which the enterprise will develop with caution cost-wise: Weights 1.2.
- Common market appeal point, which has limited appeal to customers: Weights 1.0.

After further investigation, among all of the external requirements, the responsiveness of the supply chain, quality of products or service, pollution control, protection of the ecosystem, usage of resources, fair-trading, and the promotion of corporate social responsibility in the sphere of influence are super market appeal points, with weight 1.5. Customer service, responsiveness of transportation and return, flexibility of production and transportation, recycling of water, inputs stemming from recycling, recyclable outputs, recyclable wastes, dangerous inputs, dangerous outputs, dangerous wastes, respect of biodiversity, involvement in local community, protection of private life, access to essential services, and fighting against corruption are interested market appeal points, with weight 1.2. Other external requirements are common market appeal points, with weight 1.0. Further, we have: Absolute weight = relative weight * improvement weight * market appeal point.

*Step 4. Perform a competitive assessment of internal abilities of enterprise C and determine which internal abilities to deploy.*

The competitive assessment of internal abilities includes the absolute weights, relative weights, and improvement rate. We have: Absolute weight = relative weight * improvement rate. We find that among all of the internal abilities, avoiding waste, recycling of resources, and sustainable exploitation are regarded as most important operation abilities. Furthermore, ability of computerization, cost and quality of the products, and collaborative transportation are also important capabilities in building a sustainable supply chain.

### 4.3. Suggestion and Recommendation

To further improve the overall performance in economic, environmental, and social performance, enterprise C should, firstly, have fully cognizance of its position in the industry. Through benchmarking, it is found that enterprise C still lags behind the best company in the industry in many dimensions, especially in cost of procurement, grouped sourcing, collaborative transportation, and adopting different delivery modes. Firstly, the difference between the enterprise C and the best one, in purchase cost and procurement practices, is dependent on the corporate economies of scale. A large company (which can place large orders) can usually negotiate a better supply price. Therefore, it is important for the company to expand its market share, which could help the company earn more bargaining power in procurement. At the same time, the company should optimize its procurement practice by adopting grouped sourcing, which could largely increase the work efficiency in loading and unloading, decrease loss and damage of cargo, and decrease transportation cost. Secondly, in delivery, the company could keep the transportation in-house, to deal with the delivery tasks of VIP customers within short distances, and adopt outsourcing to serve common customers at further distance. The different modes of transportation could highly control the delivery of VIP customers and, at the same time, decrease the transportation risk over long distances by hiring an outside professional transportation service. Thirdly, in distribution, the enterprise C mainly adopts direct sale. In the future, with market expansion, distributors or partners would help the company to build a stronger local commitment.

According to the absolute weights, the company should make more efforts in avoiding waste, recycling of resources, sustainable exploitation, computerization, and collaborative transportation in order to satisfy the external requirements in building the sustainable supply chain. To balance the input and output, there are several suggestions:

- **Strengthen the control of pollution:** In daily operation, choosing clean energy (instead of dirty but cheap energy) reduces air pollution. Additionally, to control dust in production, improvement of the working condition is quite necessary.
- **Increase investment to develop new technologies:** Holding the patents in resource usage, in danger control, or in ecosystem protection will easily build up the core competences and improve reputation in the industry. Therefore, it is worthwhile to increase investment in development of new technologies.
- **Implement a high level of computerization to closely work with supply chain partners:** The internal ability of computerization is the most critical capability, which could help every process in daily operation. By setting up a collaborative information system, upstream and downstream firms can work together better. With close cooperation, the receipt of orders, material preparation, and order delivery can be conducted online, which greatly improves work accuracy and efficiency. It also helps the enterprise to optimize the production plan and better supervise the operation process. The establishment of such an information system requires collaboration within the whole supply chain, and, potentially, a large investment in hardware and software. Therefore, the enterprise should share the cost with its partners, in advance.

## 5. Conclusions

Sustainable supply chains are a must-have for firms, including SMEs. Driven by the economic benefits, enterprises always pursue efficiency in every operation process, such as material management, human resources, production, and distribution. On the other hand, with the increasing deterioration of the ecological environment, the social pressures they are facing become more and more intense. Protecting the environment is not only the regulation or law which should be followed, but also a social responsibility for all corporations. Driven by social benefits, an eco-friendly corporation can maintain a good social reputation. Furthermore, building a sustainable supply chain helps to satisfy the requirements of employees, in terms of working conditions.

As the first attempt to design a sustainable supply chain for SMEs in developing countries, this paper can impel the development of SSCM in a wider field of research. This study is an original attempt in examining how SMEs can employ a relatively simple and straightforward tool, to design their sustainable supply chains. It combines QFD with AHP and benchmarking, to provide an integrated approach which could assist in determining which internal abilities to focus on and deploy. A case study of a cement manufacturing enterprise (called enterprise C in this paper) was conducted, to demonstrate the steps in implementing this approach and validate its effectiveness. In the HOQ model, the external economic, environmental, and societal requirements, and internal abilities of sourcing, production, and distribution are selected, based on interviews and a review of the related literature [56]. It is recommended that an enterprise should make more efforts in avoiding waste, recycling of resources, sustainable exploitation, computerization, procurement cost reduction, quality improvement, and collaborative transportation. Through more investment in these internal abilities, a company can improve its overall performance. Our case study in China demonstrated the approaches applicability for SMEs, in detail. More specifically, the QFD-AHP-benchmarking method can assist SME managers in implementing SSCM, following the proposed step-by-step procedure.

Our suggestions and recommendations of building a sustainable supply chain have some limitation. Firstly, the investment to build a sustainable supply chain, recommended in this paper, can be too high for the firm. In reality, cost will be the first concern in building an information system, adopting recycled resources, and collaborating with supply chain partners. In future study, if the HOQ model takes into account the required amount of investment, the recommendations would be much more implementable. Another limitation is that the performance measurements need to be changed, when applied to other industries. Finally, the scores in AHP and benchmarking in this paper are still decidedly subjective, to some extent. Therefore, our conclusions may depend on the judgment of the interviewees. Future studies should aim to employ more rigorous approaches, so that the conclusions are more robust.

## Figures and Tables

**Table 1:** The analytic hierarchy process.

Intensity of Importance	Definition	Explanation
1	Equal importance	Two activities contribute equally to the objective.
3	Weak importance of one over another	Experience and judgment slightly favor one activity over another.
5	Essential or strong importance of one over another	Experience and judgment strongly favor one activity over another.
7	Very strong or demonstrated importance of one over another	An activity is very strongly favored over another. Its dominance is demonstrated in practice.
9	Absolute importance of one over another	The evidence favoring one activity over another is of the highest possible order of affirmation.
2, 4, 6, 8	Intermediate value between adjacent scale values	For use when compromise is needed.
Reciprocals of above non-zero numbers	If the activity i has one of above non-zero numbers assigned to it when compared with activity j then j has the reciprocal value when compared to i	A reasonable assumption.

**Table 2:** Economic performance, environmental performance, and social performance.

Constructs	Factors	Reference
Economic Performance (Vachon and Klaseen, 2008) [38]	Reliability	Guansekaran, Patel, and Tirtiroglu (2001) [41]; Lynch and Cross (1991) [42]; Zhu, Sarkis, and Lai (2007) [28]
	Responsiveness	Lynch and Cross (1991) [42]; Vachon and Klassen (2008) [38]
	Flexibility	Jayaram et al. (2011) [43]
	Quality	Krajnc and Glavic (2005) [44]; Matos and Hall (2007) [29]
Environmental Performance (Murply, 1994) [39]	Environmental management	Azapagic and Perdan (2000) [45]; Darnall, Jolley, and Handfield (2008) [46]
	Use of resources	Azapagic and Perdan (2000) [45]; De Benedetto and Klemes (2009) [47]
	Pollution	De Benedetto and Klemes (2009) [47]; Krajnc and Glavic (2005) [44]; Matos and Hall (2007) [29]
	Dangerousness	Barbiroli and Raggi (2003) [48]; Zhu and Sarkis (2004) [49]
	Natural environment	Barbiroli and Raggi (2003) [48]; Michelsen, Magerholm et al. (2006) [50]
Social Performance (Drunwight, 1994) [40]	Work conditions	Azapagic and Perdan (2000) [45]; Hutchins and Sutherland (2008) [51]; O’Connor and Spanenberg (2008) [52]
	Human rights	Azapagic and Perdan (2000) [45]; Krajnc and Glavic (2005) [44]
	Societal commitment	Hutchins and Sutherland (2008) [51]; Krajnc and Glavic (2005) [44]; Matos and Hall (2007) [29]; O’Connor and Spanenberg (2008) [52]
	Customer issues	Kainuma and Tawara (2006) [53]; Veleva and Ellenbecker (2001) [54]
	Business practices	Azapagic and Perdan (2000) [45]; Castka and Balzarova (2008) [55]; Matos and Hall (2007) [29]
